# Oral intake of titanium dioxide nanoparticles affect the course and prognosis of ulcerative colitis in mice: involvement of the ROS-TXNIP-NLRP3 inflammasome pathway

**DOI:** 10.1186/s12989-023-00535-9

**Published:** 2023-06-22

**Authors:** Shumin Duan, Hongbo Wang, Yanjun Gao, Xiang Wang, Lizhi Lyu, Yun Wang

**Affiliations:** 1 Department of Occupational and Environmental Health Sciences, School of Public Health, Peking University, Beijing, 100191 P.R. China; 2 California NanoSystems Institute, University of California, Los Angeles, CA 90095 USA; 3 Beijing Key Laboratory of Toxicological Research and Risk Assessment for Food Safety, Peking University, Beijing, 100191 P.R. China

**Keywords:** Titanium dioxide nanoparticles, Oral intake, ROS-TXNIP-NLRP3 inflammasome pathway, Ulcerative colitis, Inflammatory bowel disease

## Abstract

**Background:**

Titanium dioxide (TiO_2_), no matter in nanoscale or micron sizes, has been widely used in food industry as additives for decades. Given the potential impact of TiO_2_ on the gastrointestinal epithelial and parenchymal cells, including goblet cells, the public consumers may suffer the risk of diseases caused by its widespread dissemination in food products. We therefore set out to investigate the impact of TiO_2_ NPs on the course and prognosis of ulcerative colitis by oral gavaging TiO_2_ NPs at the doses levels of 0, 30, 100, and 300 mg/kg during the induction (7 days, from day 1 to day 7) and recovery (10 days, from day 8 to day 17) phases of colitis in mice.

**Results:**

The ulcerative colitis (UC) disease model was established by administrating of 2.5% dextran sulfate sodium (DSS) solution. Our results show that TiO_2_ NPs significantly enhanced the severity of DSS-induced colitis, decreased the body weight, increased the disease activity index (DAI) and colonic mucosa damage index (CMDI) scores, shortened the colonic length, increased the inflammatory infiltration in the colon. The most significant changes occurred in the low dose (30 mg/kg) group of TiO_2_ NPs exposure during the development phase of UC and the high dose (300 mg/kg) group of TiO_2_ NPs during UC self-healing phase. Increased reactive oxygen species (ROS) level and upregulation of anti-oxidant enzymes including total superoxide dismutase (T-SOD), glutathione peroxidase (GSH-PX) and catalase (CAT), demonstrate that the TiO_2_ NP exposure has triggered oxidative stress in mice. Moreover, the upregulation of caspase-1 mRNA and increased expression of thioredoxin interacting protein (TXNIP) further demonstrate the involvement of the ROS-TXNIP-NLR family pyrin domain containing 3 (NLRP3) inflammasome pathway in aggravating the development of UC.

**Conclusion:**

Oral intake of TiO_2_ NPs could affect the course of acute colitis in exacerbating the development of UC, prolonging the UC course and inhibiting UC recovery.

**Graphical Abstract:**

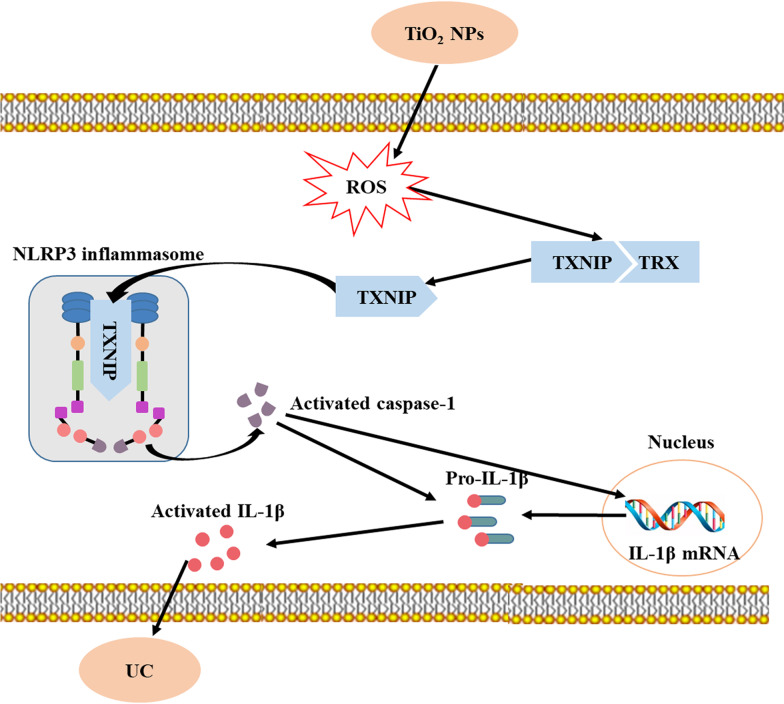

**Supplementary Information:**

The online version contains supplementary material available at 10.1186/s12989-023-00535-9.

## Highlights

- Short-term intake of TiO_2_ NPs can induce mild colitis and exacerbate colitis caused by DSS.
- Exposure to a low dose of TiO_2_ NPs exacerbated development of UC, and a high dose of TiO_2_ NPs delayed the recovery of UC.
- TiO_2_ NP exposure could trigger the activation of the antioxidant system in colitis mice.
- TiO_2_ NPs might aggravate the development of UC through the ROS-TXNIP-NLRP3 inflammasome pathway.

**Supplementary Information:**

The online version contains supplementary material available at 10.1186/s12989-023-00535-9.

## Introduction

Because of its superior whiteness, high covering power and strong whitening power, TiO_2_ is commonly used as food additives in candy, jam, chocolate, and drinks. To regulate the application of TiO_2_ in food, the US food and drug administration (FDA) has limited the weight of TiO_2_ to 1% of the total weight in food [1]. The national food safety standard in China, i.e. GB 2760–2014, also stipulated that TiO_2_ can be used as food additives in food with a limit of TiO_2_ at 10 g/kg food [2]. According to dietary exposure data from different countries, the daily intake of TiO_2_ in populations reaches approximately 1 mg/kg/d, especially in children, where it can reach up to 3 mg/kg/d [3–5]. In recent years, TiO_2_ nanoparticles (NPs) have been gradually used in food for better taste. Researchers have proven that the sizes of food additive TiO_2_ particles range from nanoscale to micronscale, of which approximately 19–39% is TiO_2_ NPs [3, 6]. Therefore, humans may expose to a good amount of TiO_2_ NPs every day through food, which arouses extensive attention on the health effect of TiO_2_ NPs as food additives.

In contrast to fine particles, nanosized particles have a smaller size range, larger specific surface area and higher surface activity, which make it easier to cross biological barriers and elicit severe biological effects. After ingestion, TiO_2_ NPs enter the gastrointestinal tract (GIT) and interact with the gut directly. Meanwhile, the gut is an important biological barrier to prevent the transport of TiO_2_ NPs. This renders the gut a major target organ of ingested TiO_2_ NPs. It has been reported that ingested TiO_2_ NPs can be absorbed by microfold cells (M cells) covering the top of Peyer’s patches. Moreover, TiO_2_ NPs can also enter and accumulate in the mesenteric lymph nodes to further affect the intestines [7–9]. It is also shown that the ingestion of TiO_2_ [10] or TiO_2_ NPs [11]. could induce inflammatory responses and therefore increased proinflammatory cytokine levels in the colon or small intestine. When the gut is in an abnormal state, more TiO_2_ NPs could be passing through the gut barrier and causing more severe damages.

Inflammatory bowel disease (IBD) is a chronic, recurrent, nonspecific and common intestinal disease that consists of two conditions, i.e. ulcerative colitis (UC) and Crohn’s disease (CD) [12, 13]. It affects all age groups with adolescents and young adults at highest risk of diagnosis. It is normally reported in North America, Europe, Australia and other western countries [14]. However, in China, with the improvement of life quality and changes in lifestyle, the morbidity has been gradually increasing. The exact cause of IBD is still unknown, but it is the result of a weakened immune system and the interaction of the host’s immunity, genetic background, and microorganisms etc. It is worth noting that the particles ingested through the diet, such as TiO_2,_ are known to be a common risk factor for IBD [15]. Several studies report that black particles composed of TiO_2_ and aluminosilicate could be seen aggregating in the Peyer’s patches of children with IBD, especially in children with UC [16]. The Ti content in blood was higher in patients at the active UC stage than in healthy volunteers [17]. All these suggest that TiO_2_ ingested through the diet plays important roles in the process of IBD, especially in UC.

To date, studies regarding the impact of TiO_2_ NPs on UC are limited. Chen et al. reported that no colitis-like symptoms or apparent disturbance of gut microbiota was found in mice ingested TiO_2_ NPs [18]. Ruiz et al. reported that intragastric administration of TiO_2_ NPs could worsen intestinal inflammation and exacerbate dextran sulfate sodium (DSS)-induced acute UC through the activation of the NLR family pyrin domain containing 3 (NLRP3) inflammasome [17]. However, the exact mechanism of whether TiO_2_ NPs affect UC through the activation of NLRP3 inflammasome or by different mechanisms on different stages of UC is still not clear. Existing studies mostly explained the induction or aggravation of inflammation by TiO_2_ NPs from the perspective of oxidative stress. Exposure to TiO_2_ NPs can induce reactive oxygen species (ROS) production and influence the expression of enzymes associated with oxidative stress, including glutathione peroxidase (GSH-PX) and total superoxide dismutase (T-SOD), etc., in colorectal cancer cell and liver cell lines, or in the kidneys and intestines in vivo [19–24]. Oxidative stress and ROS generation are thought to be pathways involved in the activation of NLRP3 inflammasome [25]. There is a few studies have pointed to the existence of the ROS-TXNIP-NLRP3 inflammasome pathway in the liver, while demonstrating that the silencing of TXNIP could prevent the activation of NLRP3 inflammasome during oxidative stress [26, 27]. Therefore, we hypothesize that the ROS-TXNIP-NLRP3 inflammasome pathway also plays an important role in the intestine and is involved in the TiO_2_ NPs induced ulcerative colitis (UC).

**Fig. 1:**
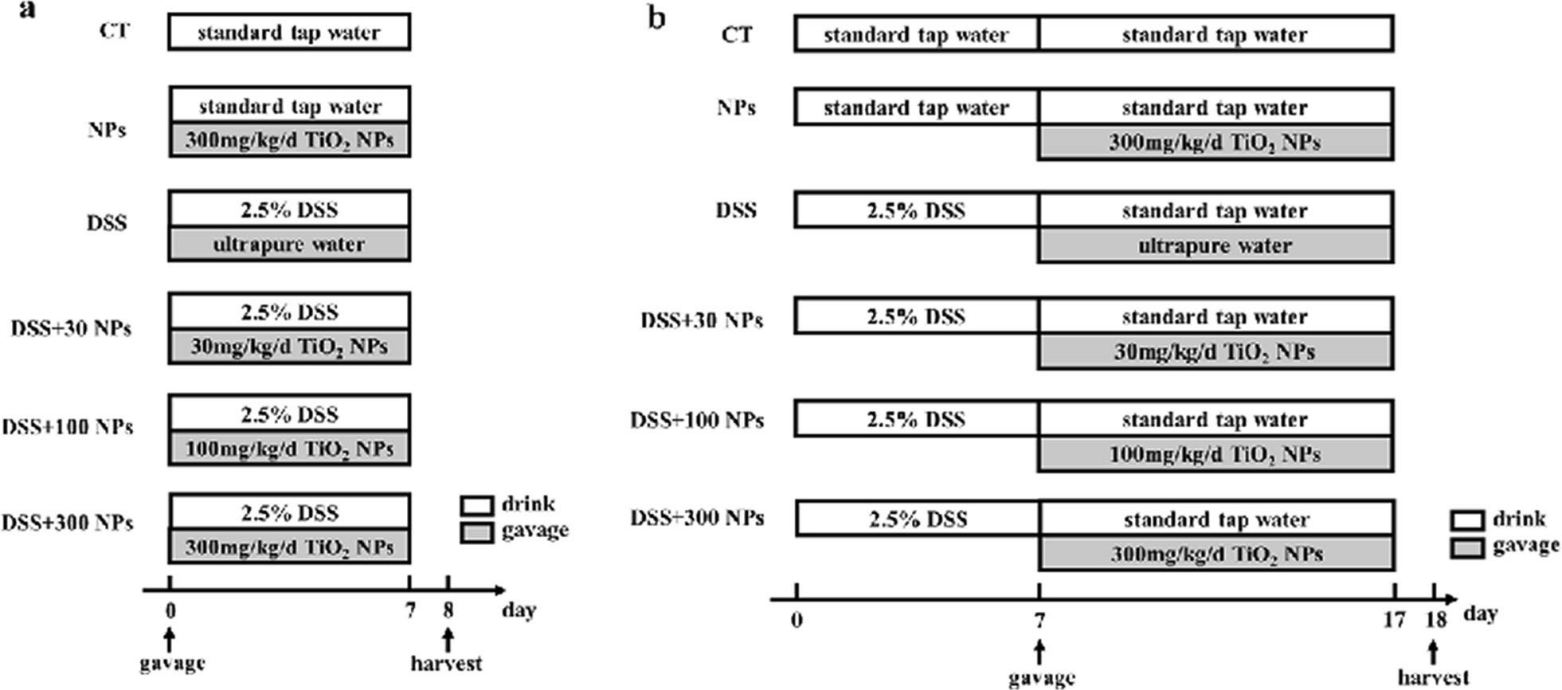
Schedule of exposure in the experiment of UC developing (**a**) and UC self-healing (**b**). The operation in white grids was carried out through drinking and the operation in gray grids was carried out through gavage. CT, control group; NPs, 300 mg/kg/d TiO_2_ NPs treated group; DSS, 2.5% DSS treated group; DSS + 30/100/300 NPs, 2.5% DSS + 30/100/300 mg/kg/d TiO_2_ NPs treated group

This study established an acute mouse ulcerative colitis (UC) model by using dextran sulfate sodium (DSS). The animals were exposed to TiO_2_ NPs at varying doses by gavage during the development or self-healing phase of acute UC (Fig. 1). We aimed to observe the influence of TiO_2_ NPs on the process of UC at different stages and explore the ROS-TXNIP-NLRP3 inflammasome pathway in the TiO_2_ NPs induced ulcerative colitis (UC).

## Results and discussion

### Establishment of DSS-induced ulcerative colitis mouse models at development or self-healing phases

A commonly used method to establish ulcerative colitis is by administering of dextran sulfate sodium (DSS) to animals [28–30]. Practically, it induces injuries to the intestinal epithelium, resulting in exposure of the lamina propria (LP) and submucosal compartment to antigens and bacteria, triggering inflammatory responses. We chose this method also because it is closer to human UC in morphology and symptoms. Balb/c mice were exposed to the 2.5% DSS solution freely for 7 continuous days for the establishment of acute UC model. Then the DSS solution was replaced with standard tap water for another 10 days for its self-healing.

**Fig. 2:**
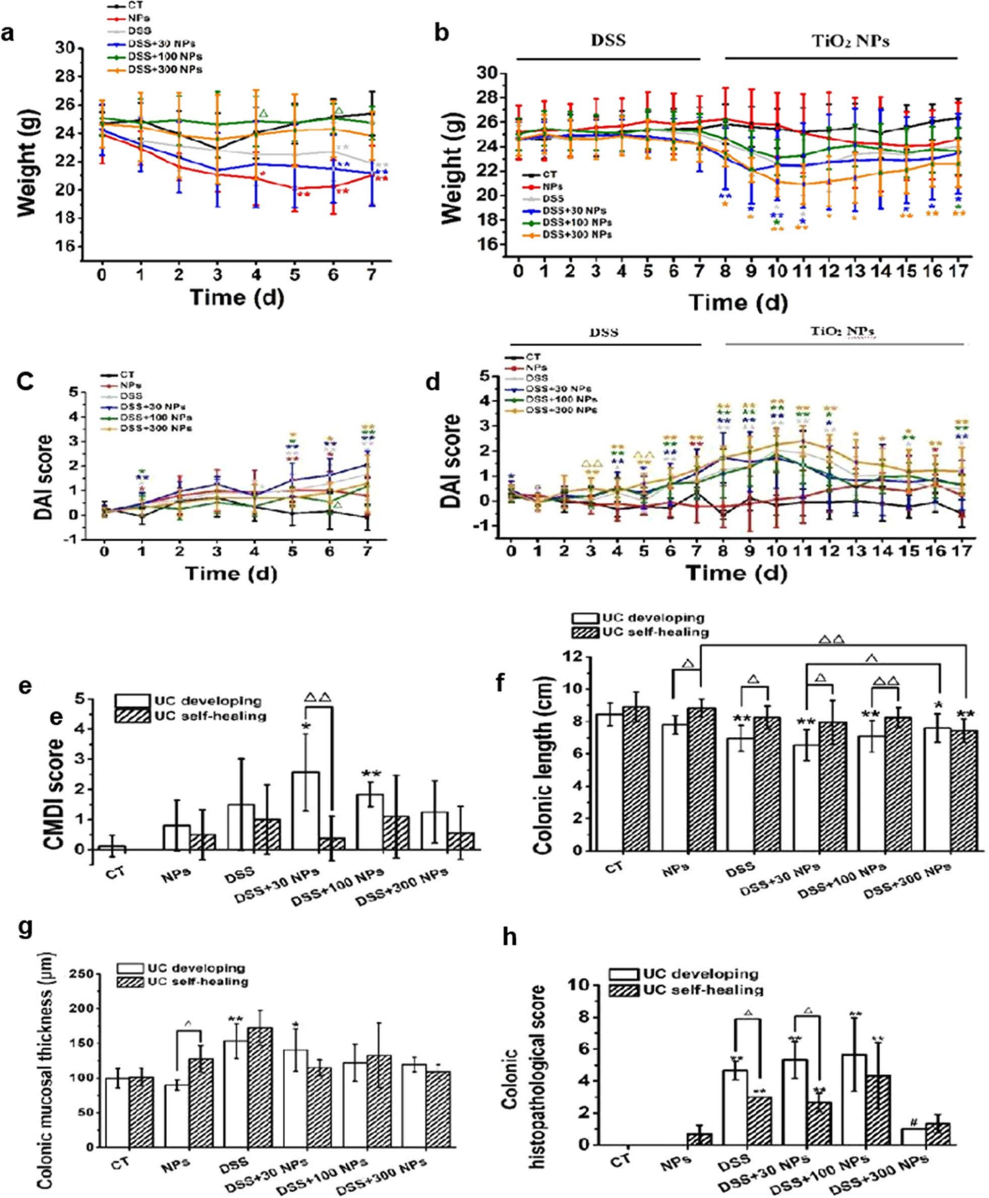
Change of mice colitis activity after treatment with TiO_2_ NPs. Change of body weight of mice in the experiment of UC developing (**a**) and UC self-healing (**b**), and change of the DAI score in the experiment of UC developing (**c**) and UC self-healing (**d**) (*n* = 10, $\overline{x }$±*s*). *p < 0.05, **p < 0.01, compared with CT group; Δ*P* < 0.05, ΔΔ*P* < 0.01, compared with DSS group. The CMDI score (**e**, *n* = 10), colonic length (**f**, *n* = 10), colonic mucosal thickness (**g**, *n* = 3), and colonic histopathological score (**h**, *n* = 3) in the experiment of UC developing and UC self-healing ($\overline{x }$ ± *s*). *p < 0.05, **p < 0.01, compared with CT group; Δ*P* < 0.05, ΔΔ*P* < 0.01, comparison between two groups

**Fig. 3:**
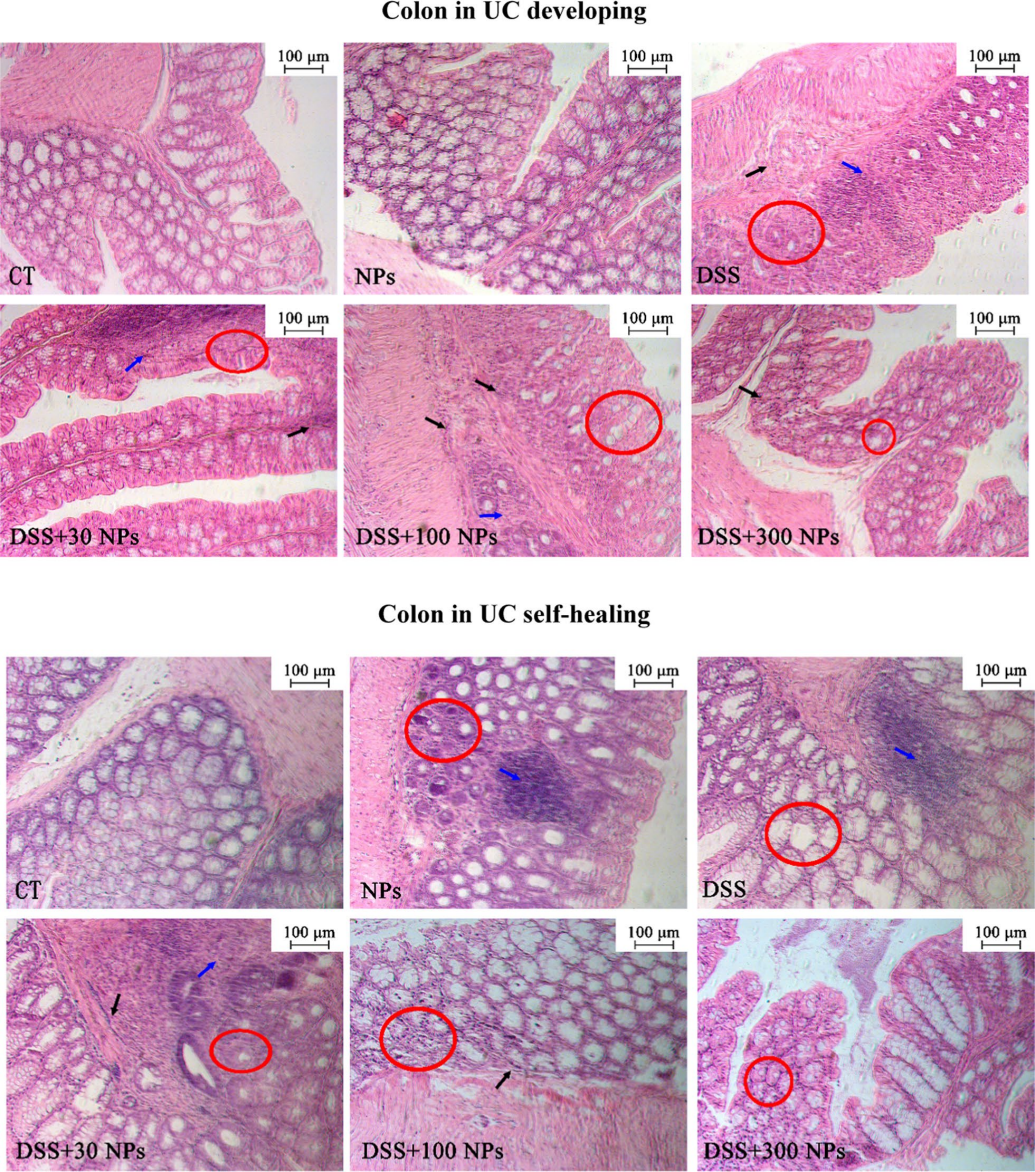
Histopathological changes in colon of mice in the experiment of UC developing and UC self-healing. Red circles indicate areas of goblet cells loss; black arrows indicate inflammatory cell infiltration; blue arrows indicate loss of crypt

**Fig. 4:**
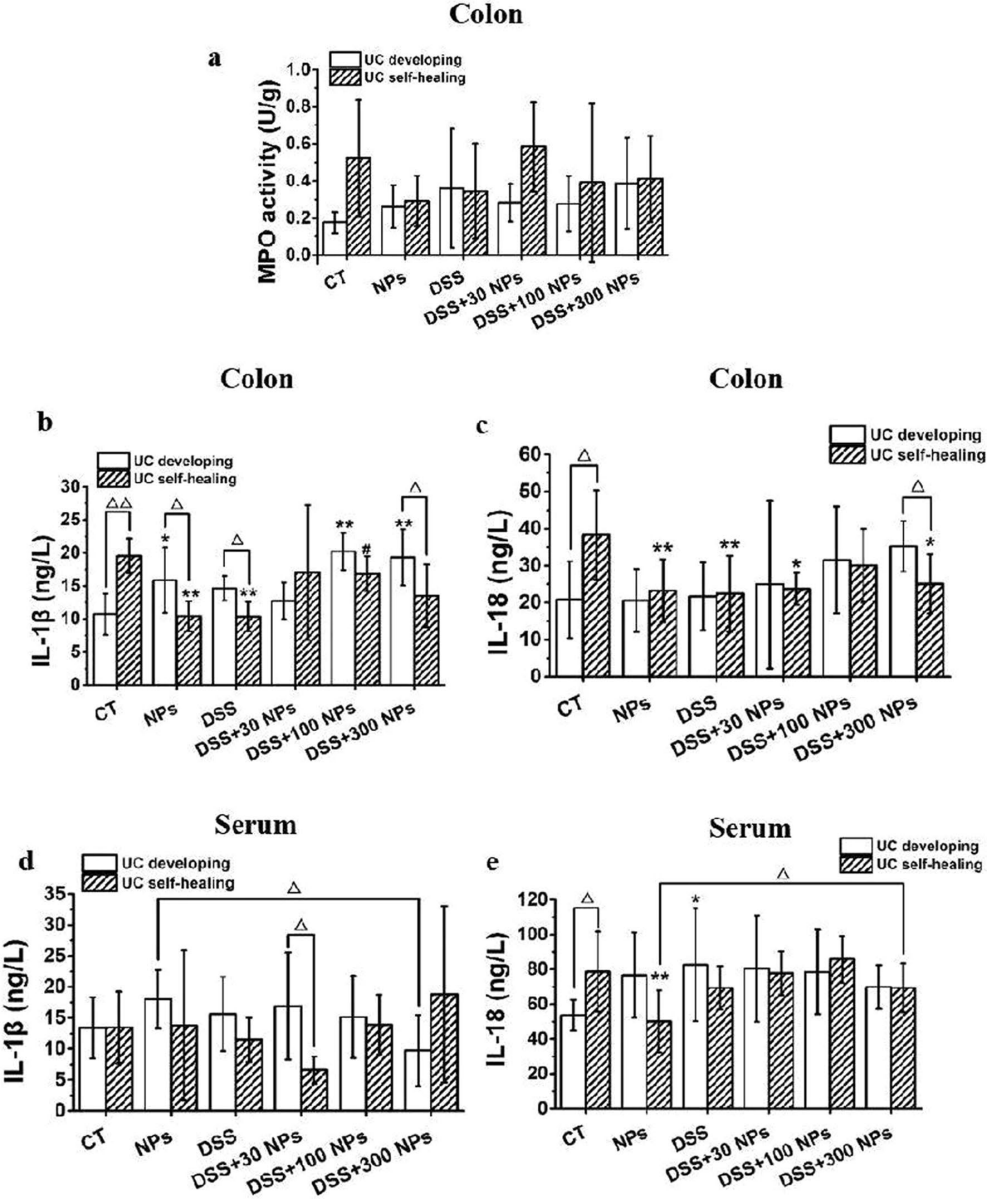
Inflammatory response level in colitis mice. Colon MPO activity (**a**) and cytokines level of colon (**b**, **c**) and serum (**d**, **e**) in the experiment of UC developing and UC self-healing (*n* = 6, $\overline{x }$ ± *s*). **a**–**c** MPO activity, IL-1β expression and IL-18 expression in colon; d, e, IL-1β and IL-18 expression in serum. *p < 0.05, **p < 0.01, compared with CT group; #*P* < 0.05, compared with DSS group; Δ*P* < 0.05, ΔΔ*P* < 0.01, comparison between two groups indicated

In the UC development phase, the mice that were given DSS solution began to exhibit characteristics including dull hair, loose feces, apathetic, and decreased activity from the 3rd exposure day and showed hematochezia and weak adhesion caused by hematochezia on the 6th day. The DSS-induced acute UC model had a low mortality rate, and the existing studies reported that the mortality varied from 0 to 40% [31–33]. In this study, partial death occurred on the 7th day, and the mortality rate of mice in the DSS-induced colitis model group was as low as 10% (Additional file 1: Table S1). Meanwhile, the body weight of mice treated with DSS also showed a significant decrease with prolonged treatment time compared with the control (CT) group (Fig. 2a). As shown in Fig. 2c, the DAI score of mice in the DSS group continuously increased and was significantly higher than that of the control group on days 1, 4 and 7. Changes in behavior, appearance, body weight and DAI score visually showed that disease occurred in mice. Then, mice were harvested and more indexes were tested. In the DSS group, the CMDI score showed an increasing trend, the colonic mucosal thickness and colonic histopathological score significantly increased, and colonic length was significantly shortened (Figs. 2 and 3). Further detection of inflammation showed an increasing trend of MPO activity and IL-1β level in the colon and serum, and a significant increase in IL-18 level in serum (Fig. 4). These characteristic’s changes [34, 35] suggested that acute UC had occurred in mice after they were given DSS solution for 7 days.

In the self-healing phase of UC, the condition of the mice was not improving at the beginning but progressively worsened after the DSS solution was replaced with standard tap water. It was shown that the mice in the DSS group continued to lose weight from the 8th to the 11th day, after which they regained weight (Fig. 2b). The DAI score continued to increase from the 8th to the 10th day, after which it began to gradually decline (Fig. 2d). However, lower mouse body weight and higher DAI score was still found in the DSS group than in the CT group in the self-healing phase of UC (Fig. 2). These dynamic trends indicated that the inflammation of mice continued to increase after DSS administration was stopped and then gradually recovered. On day 18, the CMDI score, colonic mucosal thickness, colonic length, IL-1β, and IL-18 in the serum of mice in the DSS group were not significantly different from those in the CT group (Fig. 2 and 4), meaning that acute UC was in the rehabilitation stage. While the CMDI score and colonic mucosal thickness in the DSS group showed a trend of increase, the colonic histopathological score was significantly high, and colonic length showed a trend of decrease, so we speculated that acute UC in mice had not fully recovered (Fig. 2).

### Oral intake of TiO_2_ NPs caused mild intestinal inflammation in mice

**Fig. 5:**
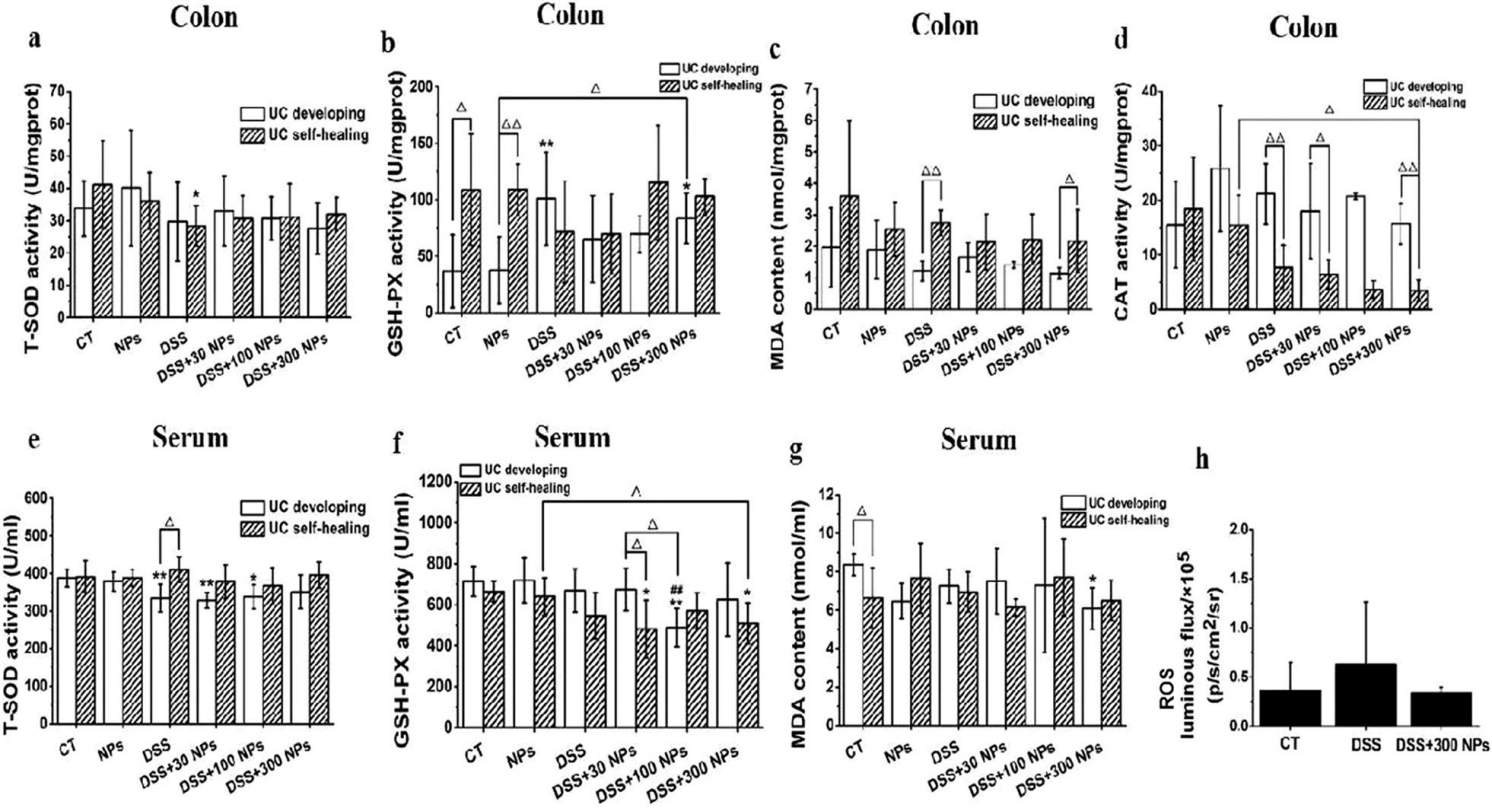
Oxidative stress in colitis mice. Antioxidase activity level of colon and serum in the experiment of UC developing and UC self-healing (*n* = 6, $\overline{x }$ ± *s*). T-SOD (**a**), GSH-PX activities (**b**), MDA content (**c**) and CAT activity (**d**) in colon in turn; T-SOD (**e**), GSH-PX activities (**f**), MDA content (**g**) in serum in turn. In vivo detection of ROS luminous flux in mice abdominal in the experiment of UC developing (**h**) (*n* = 3, $\overline{x }$ ± *s*). *p < 0.05, **p < 0.01, compared with CT group; ##*P* < 0.01, compared with DSS group; Δ*P* < 0.05, ΔΔ*P* < 0.01, comparison between two groups indicated

In this study, we established NP groups to study the influence of ingested TiO_2_ NPs. Nine-week-old mice were gavaged with 300 mg/kg/d TiO_2_ NPs for 7 continuous days in the UC development experiment, and 10-week-old mice were administered 300 mg/kg/d TiO_2_ NPs for 10 continuous days in the UC self-healing experiment. The results showed significantly decreased body weight of mice with the exposure time of TiO_2_ NPs, the obviously high DAI score and colon IL-1β level, the increasing trend of CMDI score, colonic mucosal thickness, colonic histopathological score, colon MPO, T-SOD, and CAT activity, serum IL-1β, IL 18, and MDA level, and the decreasing trend of colonic length in mice treated with TiO_2_ NPs, while these changes were less apparent than in the DSS group (Figs. 2, 3, 4, 5). This suggests that short-term oral administration of TiO_2_ NPs triggered the intestinal inflammatory reaction, but the symptoms were milder than DSS-induced colitis.

Some studies reported that short-term exposure (24 h) to different doses of TiO_2_ NPs (10 μg/ml or 5, 20, 100 μg/ml) on human colorectal adenocarcinoma Caco-2 cells could induce high release of proinflammatory cytokines IL-8, IL-1β, and IL-18 [17, 36]. When given 100 mg/kg/d TiO_2_ NPs by gavage for 10 days, the levels of various inflammatory cytokines in the mouse jejunum and ileum were elevated, including IL-1β, IL-6, IL-8, IL-12, IL-4, IL-13, tumor necrosis factor γ (TNF-γ), and IFN-γ, suggesting the induction of small intestinal inflammation [11]. After 30 days of continuous intragastric administration of 10, 50 and 100 mg/kg bw/day TiO_2_ NPs, the activities of SOD, CAT, GSH-Px, and GSH decreased, the content of MDA increased, the intestinal mucosa and recess structure was damaged, and the intestinal IL-1β level increased significantly, indicating that TiO_2_ NPs induced intestinal oxidative stress and inflammatory response in rats [24]. After administration of 10 mg/kg bw/day E171 TiO_2_ by gavage for 100 days, the cytokines TNF-α, IL-1β, IL-18, IL-10, IL-8 and IL-6 increased in the colonic mucosa of rats, suggesting that food grade TiO_2_ induced slight inflammation of the rat colon [10]. Combined with the above research reports and the results of this study, it can be determined that oral intake of TiO_2_ NPs will trigger an intestinal inflammatory response and then play a role in the occurrence and development of IBD.

### TiO_2_ NPs aggravated the development of UC and slowed down the self-healing process of UC

To study the effect of TiO_2_ NPs on the course of UC, we gave mice 30 (low dose), 100 (medium dose), and 300 (high dose) mg/kg/d TiO_2_ NPs through gavage during the development and self-healing of acute UC induced by DSS. The results showed that TiO_2_ NPs changed the severity of UC caused by DSS in mice, resulting in significant weight loss, an increase in DAI and CMDI scores, a shortened colon, inflammatory infiltration in the colon, and increased expression of IL-1β and IL-18 (Figs. 2, 3, 4). However, different doses of TiO_2_ NPs caused different changes in UC mice. During UC development, UC mice treated with a low dose of TiO_2_ NPs had the lowest weight, highest DAI and CMDI scores, and the shortest colon length; UC mice treated with a medium dose of TiO_2_ NPs had the highest colonic histopathological score and highest level of IL-1β in the colon; and UC mice treated with a high dose of TiO_2_ NPs had the highest MPO activity and IL-18 level in the colon. During UC self-healing, UC mice treated with a high dose of TiO_2_ NPs had the lowest weight, highest DAI score, shortest colon length and highest IL-1β level in serum; UC mice treated with a medium dose of TiO_2_ NPs had the highest colonic histopathological score and CMDI score and the highest levels of IL-1β and IL-18 in the colon; and UC mice treated with a low dose of TiO_2_ NPs had the highest MPO activity (Figs. 2, 3, 4). It was not hard to see that TiO_2_ NPs influenced the process of UC developing and UC self-healing, performing as low dose of TiO_2_ NPs aggravated the developing of UC and high dose of TiO_2_ NPs prolonged the course of UC, slowed the UC self-healing.

Few studies have reported the effect of TiO_2_ NPs on colitis. After UC mice induced by DSS were gavaged with 50 and 500 mg/kg/d TiO_2_ NPs for 7 days, colonic length decreased, histopathological score increased, and inflammatory infiltration became more severe, indicating that TiO_2_ NPs exacerbated colitis. The toxicity of 500 mg/kg/d TiO_2_ NPs was basically consistent with that of 50 mg/kg/d TiO_2_ NPs and even weaker than that of 50 mg/kg/d TiO_2_ NPs [17]. Another group of researchers induced chronic colitis in mice by giving mice DSS solution 1 week every 3 weeks and looping 3 times, while the mice were given 0.1% (mass fraction) TiO_2_ NPs through fodder. They found that TiO_2_ NPs caused weight loss in mice, colon shortening and disorder of intestinal immunity [37]. Our results were consistent with these studies showing that TiO_2_ NPs aggravated DSS-induced UC, but different exposure doses may have different effects.

The NP group in our study showed that exposure to a high dose of TiO_2_ NPs could induce intestinal inflammation in mice, which may play a role in the occurrence and development of IBD. UC mice treated with a high dose of TiO_2_ NPs did not present a noticeable exacerbation of the development of UC, possibly because a high dose of TiO_2_ NPs formed aggregates by adsorbing many intestinal contents, and then the aggregates covered the intestinal surface, preventing contact between DSS and the intestine. In addition, the aggregation of TiO_2_ NP resulted in less interaction with the intestinal wall. The large hydrodynamic diameters of the TiO_2_ NPs in ultrapure water, AGJ and AIJ suggest that they would aggregate in the animal gastrointestinal track. Even so, inflammation in the DSS + 300 NP group was still heavier than that in the NP group. In UC self-healing, acute UC was induced, and ulceration formed in the colon before TiO_2_ NP treatment. Exposure to high-dose TiO_2_ NPs caused more TiO_2_ NPs to enter the intestinal epithelium and had a direct impact on intestinal injury, thus prolonging the recovery time of UC.

### TiO_2_ NPs trigger the oxidative stress and impact the antioxidant system in UC mice

Oxidative stress is an important mechanism underlying the biological effects of nanomaterials. To investigate the change in oxidative stress in UC mice caused by TiO_2_ NPs, we tested ROS and MDA content, T-SOD, GSH-PX, and CAT activities in colonic homogenate, T-SOD and GSH-PX activities and MDA content in serum (Fig. 5). Compared with the CT group, the DSS groups showed significantly increased colon GSH-PX activity and obviously decreased serum T-SOD activity during UC development and obviously decreased colon T-SOD activity during UC self-healing. The DSS + NP groups presented significantly increased colon GSH-PX activity and obviously decreased serum T-SOD and GSH-PX activity during UC development and obviously decreased serum GSH-PX activity during UC self-healing. Compared with DSS groups, DSS + NPs groups displayed slightly increased T-SOD activity and MDA content and decreased ROS level, GSH-PX and CAT activities in colon and serum during the UC developing, and DSS + NPs groups displayed increased T-SOD, GSH-PX activities in colon, decreased CAT activity and MDA content in colon, and decreased T-SOD, GSH-PX activities and MDA content in serum during the UC self-healing, but these differences were not statistically significant. These results showed that colitis mice were in a state of oxidative stress, and TiO_2_ NP exposure could further trigger the activation of the antioxidant system but did not significantly change the level of oxidative stress in colitis mice.

Oxidative and antioxidant systems are in a state of homeostasis to maintain the normal physiological functions in the human body. This status can be easily affected by various factors, such as infection, inflammation, and invasion of xenobiotics, which would lead to excessive ROS and oxidative stress [38]. The change in MDA content and antioxidant activities, including T-SOD, GSH-PX, and CAT, could reflect the state of oxidative stress in organisms [24]. MDA is the peroxidation product of polyunsaturated fatty acids, which are usually used to detect lipid peroxidation [39]. T-SOD is an antioxidant metal enzyme that mainly catalyzes superoxide to generate oxygen and hydrogen peroxide, protecting the body from oxidative stress [40]. GSH-PX is a peroxide decomposition enzyme that oxidizes glutathione (GSH) to oxidized glutathione (GSSG) by consuming ROS to maintain the normal function of cells. CAT can reduce hydrogen peroxide to water and oxygen to relieve the damage of oxygen free radicals to the body. Therefore, these antioxidant enzymes are activated to scavenge ROS and reduce oxidative stress when ROS are produced in excess in the body. It should be noted that the body will show two states in response to oxidative stress: one is to compensate for upregulating the activity of antioxidant enzymes to actively cope with oxidative stress, and the other is to deplete antioxidant enzymes to eliminate ROS, resulting in the decline of antioxidant enzyme activity [41]. Therefore, in our study, the increase in GSH-PX activity and decrease in T-SOD activity in the colon and serum in the DSS and DSS + NP groups were all the results of the body’s response to oxidative stress. GSH-PX was upregulated to actively scavenge oxygen free radicals, and T-SOD was consumed too much when scavenging oxygen free radicals. Furthermore, there were no significant differences in the antioxidant enzyme activity and MDA content between the DSS + NP groups and DSS group, suggesting that the oxidative stress level changed slightly after ingestion of TiO_2_ NPs.

### TXNIP and the NLRP3 inflammasome involved in the oxidative stress caused by TiO_2_ NPs

**Fig. 6:**
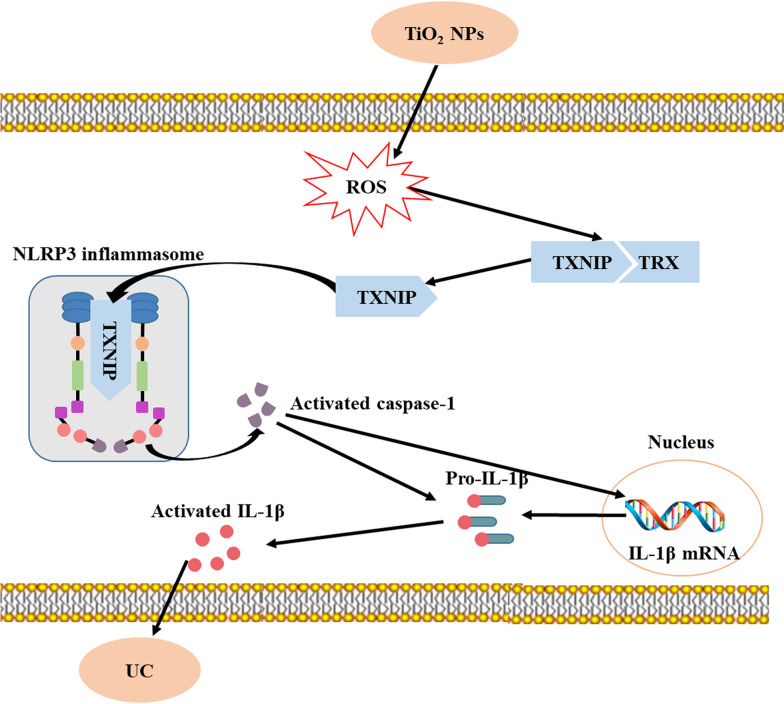
Possible mechanisms of the effect of TiO_2_ NPs on UC via ROS-TXNIP-NLRP3 inflammasome pathway

**Fig. 7:**
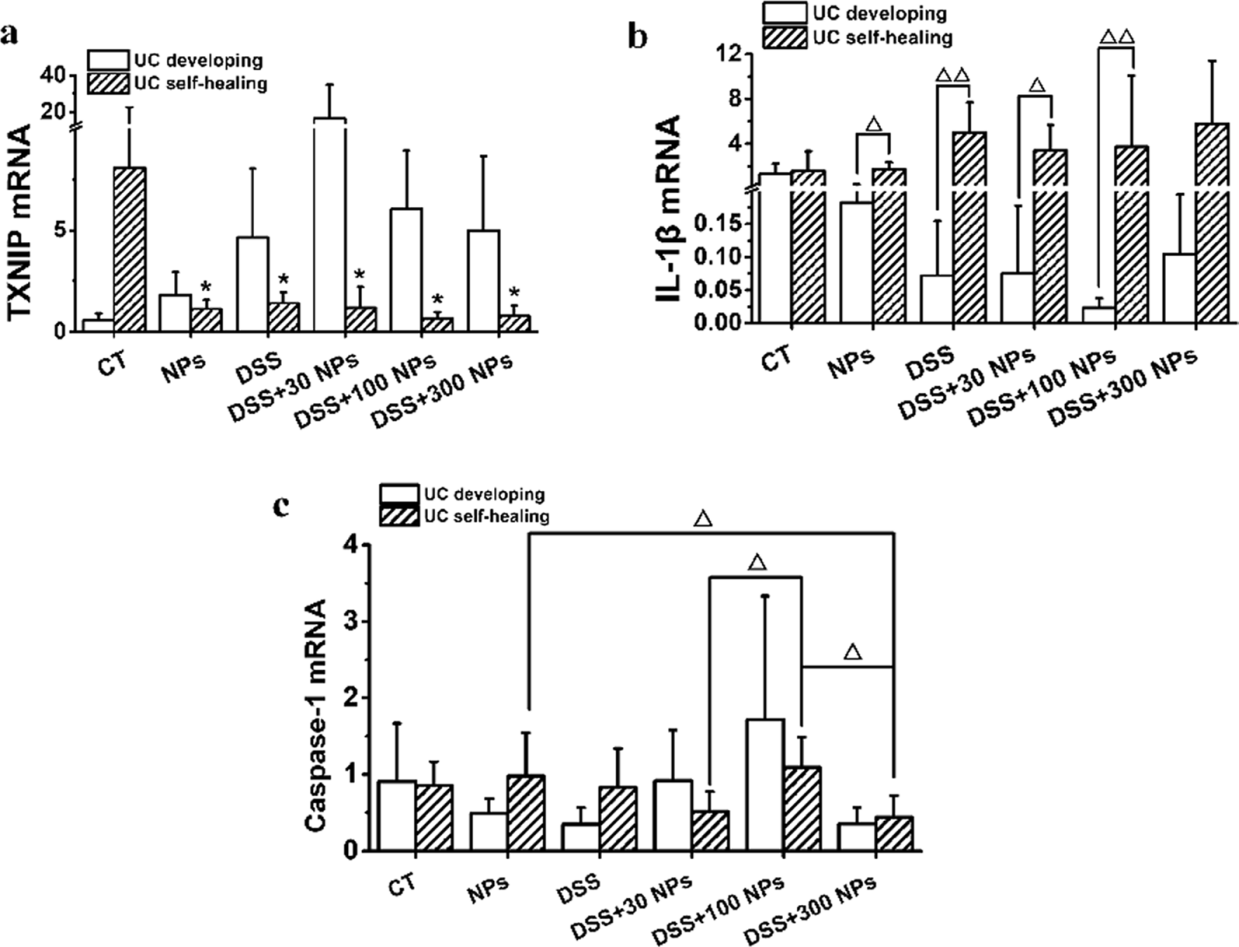
TXNIP, caspase-1, IL-1β mRNA expression of colon in the experiment of UC developing and UC self-healing (*n* = 6, $\overline{x }$± *s*). * p < 0.05, compared with CT group; Δ*P* < 0.05, ΔΔ*P* < 0.01, comparison between two groups

To further reveal the possible mechanism of the effect of TiO_2_ NPs on the course of UC in mice, we proposed the hypothesis that TiO_2_ NPs affected UC through the ROS-TXNIP-NLRP3 inflammasome pathway (Fig. 6) and detected the mRNA expression of TXNIP, caspase-1, and IL-1β (Fig. 7). In the experiment of UC development, the increase in TXNIP and caspase-1 mRNA expression in the DSS + NP groups compared with the DSS group suggested that TiO_2_ NPs might aggravate the development of UC through the ROS-TXNIP-NLRP3 inflammasome pathway. In the UC self-healing experiment, the TXNIP and caspase-1 mRNA levels were decreased in the DSS + NP groups compared with the DSS group, suggesting that TiO_2_ NPs did not depend on the ROS-TXNIP-NLRP3 inflammasome pathway to delay UC self-healing.

The NLRP3 inflammasome, which belongs to the NLR family, is a protein complex composed of sensor NLRP3 protein, adapter ASC, and effector caspase-1. The NLRP3 inflammasome can react to stimulants such as particulates, toxins, and microbial receptor agonists, and then the caspase-1 precursor is activated by ASC to caspase-1, which further converts IL-1β and IL-18 precursors into activated IL-1β and IL-18, thereby promoting cytokine release [42, 43]. TiO_2_ NPs, as exogenous particles, can also activate the NLRP3 inflammasome, which has been proven by studies [17, 24, 43–45]. Rats were gavaged with 10, 50, or 100 mg/kg/d TiO_2_ NPs for 30 days, and the mRNA expression of NLRP3, caspase-1, and IL-1β was significantly increased and the NLRP3 inflammasome was activated by TiO_2_ NPs [24] Activation of the NLRP3 inflammasome is essential in the development of UC [43–45], and TiO_2_ NPs do not affect DSS-induced colitis in NLRP3-deficient mice, meaning that TiO_2_ NPs aggravate DSS-induced enteritis through the activation of the NLRP3 inflammasome [17]. In this study, the increase in caspase-1 mRNA expression and colonic IL-1β levels in UC mice treated with TiO_2_ NPs further proved that NLRP3 inflammasome activation plays an important role in the occurrence and development of UC exacerbated by TiO_2_ NPs. Consistent with expectations, IL-1β mRNA expression had no significant change, as upon NLRP3 inflammasome activation caspase-1 cleaves pro-IL-1β to IL-1β, not requiring regulation at the transcriptional level.

TXNIP is an endogenous inhibitor and regulator of thioredoxin (TRX). TXNIP binds with TRX in general, but under conditions of oxidative stress, TXNIP will be separated from TRX and combine with the NLRP3 inflammasome. Therefore, the NLRP3 inflammasome is activated, and free TRX can reduce oxidized proteins and play an antioxidant role [46]. So just like response of body to oxidative stress, increased TXNIP may indicate generation of oxidative stress, or may indicate fight of body to oxidation. This could explain the significantly increase of TXNIP during UC self-healing. Studies have proven the activation of NLRP3 by TXNIP. For example, mouse N9 microglia were treated with 1 μM cortisol for 5 days, and then the TXNIP protein level, a combination of TXNIP and NLRP3 significantly increased, the level of caspase-1 precursor protein decreased significantly, and the level of activated caspase-1 and IL-1β increased significantly in cells. The activation of caspase-1 and IL-1β was inhibited when TXNIP was knocked off in N9 cells, suggesting that TXNIP had an important effect on the activation of the NLRP3 inflammasome [47]. In addition, the ROS-TXNIP-NLRP3 inflammasome axis has also been reported to exist in other organs, such as kidney, liver, neurocyte, and periodontal membrane cells [26, 48–50]. In our study, the increase in TXNIP and caspase-1 mRNA expression in the colon during UC development also stated that TXNIP could activate the NLRP3 inflammasome. However, the activation of the NLRP3 inflammasome is affected by many factors, and the protein level of each component in the TXNIP and NLRP3 inflammasome was not measured in this study, nor were gene knockout validation studies conducted, so whether TXNIP plays a key role in the activation of the NLRP3 inflammasome in mice with colitis was not clear until further study.

## Conclusion

This study found that oral intake of TiO_2_ NPs could aggravate the development of DSS-induced UC in mice, prolong the course of UC, and slow the self-healing process of UC. However, in different stages of UC, the effect of TiO_2_ NPs on colitis showed different dose–response relationships, manifesting as aggravation of UC development by low-dose TiO_2_ NPs and delay of UC self-healing by high-dose TiO_2_ NPs. Considering that the effect of TiO_2_ NPs on colitis is complex, it is recommended that IBD patients try to avoid using food containing TiO_2_ NPs.

## Material and Methods

### TiO_2_ NPs characterization

**Fig. 8:**
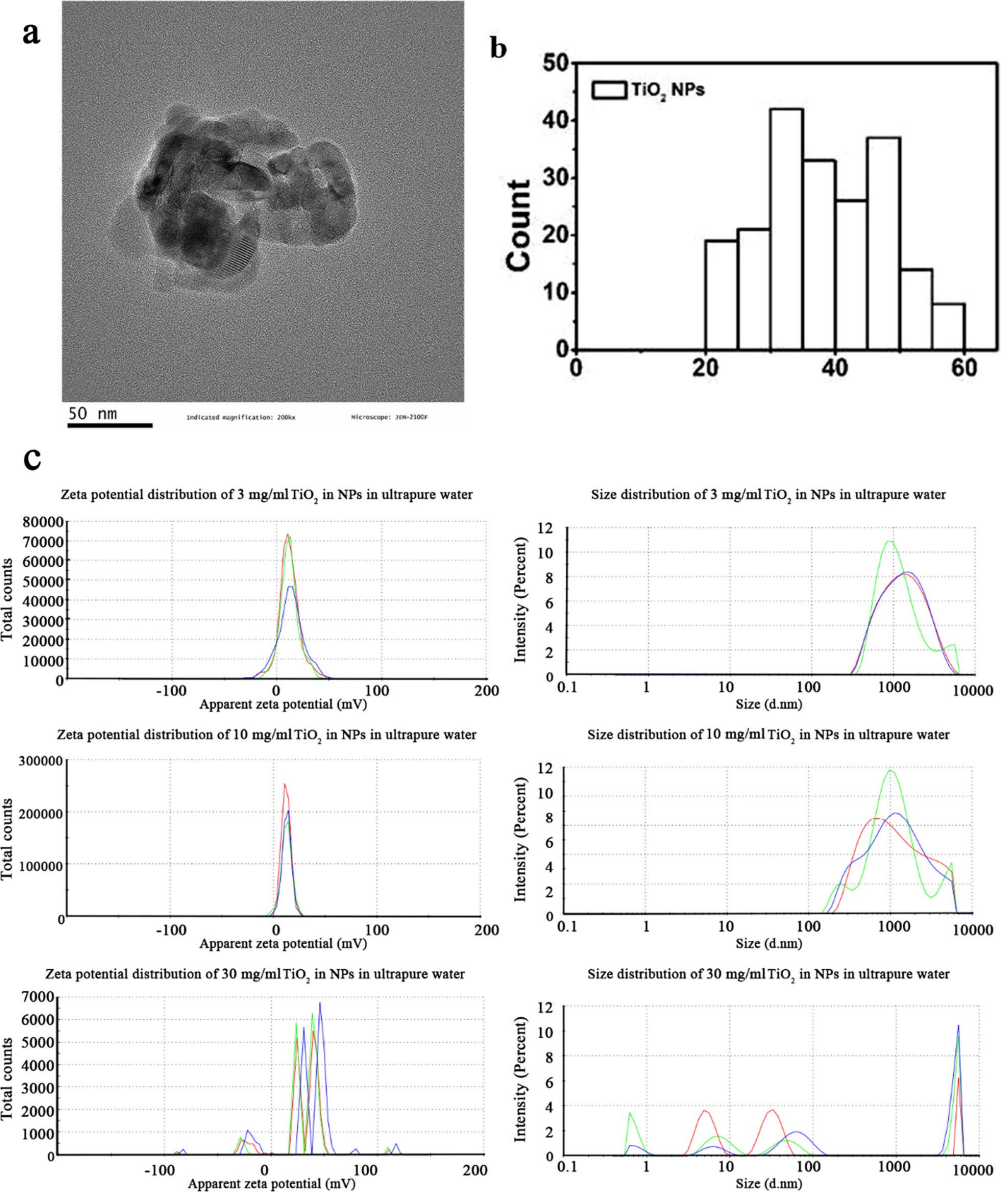
Characterization of TiO_2_ NPs. The shape, size (**a**) and particles size distribution (**b**) of TiO_2_ NPs based on TEM. The zeta potential and hydrodynamic diameter of 3, 10 and 30 mg/ml TiO_2_ NPs in ultrapure water (**c**) tested at 0 h post sonication. The red, green and blue line represents three parallel detections

TiO_2_ NPs were purchased from Shanghai Yunfu Nanotechnology Co. Ltd, China. The characterization of TiO_2_ NPs was performed in our previous studies [51–53]. Briefly, TiO_2_ NPs were dispersed in absolute ethyl alcohol and ultrasound for 15 min (SB-50D, 20 kHz, 50W). Two drops of the suspension were put onto carbon-coated transmission electron microscopy (TEM, JEM-2100F, JEOL, Japan) grids prior to image capture. TEM was used to determine the primary size and shape of TiO_2_ NPs. X-ray powder diffractometry (XRD, X’Pert Pro, PANalytical) was used to detect the crystal structure of TiO_2_ NPs. The Brunauer–Emmett–Teller (BET) method (Autosorb-iQ2-MP, Malvern Panalytical) was used to measure the specific surface areas of TiO_2_ NPs. Inductively coupled plasma–mass spectrometry (ICP–MS, Thermo Elemental X7, Thermo Electron Corporation, USA) was used to determine the purity of TiO_2_ NPs. As described in our previous study [52], the artificial gastric juice (AGJ) (pH 1.2) was prepared with 10 g/L pepsin (3800 units/mg) and 45 mmol/L HCl; the artificial intestinal juice (AIJ) (pH = 6.8) was constituted by 10 g/L trypsin and 6.8 g/L KH_2_PO_4_. TiO_2_ NPs were suspended in ultrapure water, AGJ or AIJ respectively and ultrasonicated for 15 min (SB-50D, 20 kHz, 50W), then the hydrodynamic diameter and Zeta-potential were tested at 0–2 h post sonication using ZetaSizer Nano ZS90 (Malvern Panalytical, UK). The results showed that TiO_2_ NPs were in anatase form and nearly spherical in shape with an average primary diameter of 33.6 ± 11.5 nm (Fig. 8). The purity of TiO_2_ NPs was higher than 99.95%. The specific surface area was 61.87 m^2^/g in TiO_2_ NPs. The hydrodynamic diameters, polydispersity index (PDI) and zeta potential of TiO_2_ suspension were changed with the interval time between tests after ultrasonic treatment (Additional file 1: Table S2, Figure S1). Testing at 0 h post sonication, the PDI of TiO_2_ in ultrapure water, AGJ and AIJ increased with the increase of the concentration of TiO_2_ NPs (Additional file 1: Table S2, Fig. 8), indicating that low dose of TiO_2_ NPs has good dispersion stability after sonication. The hydrodynamic diameters of 3 mg/ml TiO_2_ NPs were 1079 ± 23 nm in ultrapure water, 2298 ± 24 nm in AGJ, and 3207 ± 103 nm in AIJ, respectively (Additional file 1: Table S2). The zeta potentials of 3 mg/ml TiO_2_ NPs were 12.3 ± 0.69 mV in ultrapure water, 2.77 ± 1.11 mV in AGJ, and − 11.4 ± 1.1 mV in AIJ, respectively (Additional file 1: Table S2).

### Establishment of acute UC mice model

A 2.5% DSS solution was prepared by dissolving 2.5 g DSS (molecular weight 36,000–50,000, MP Biomedicals, USA) in 100 mL ultrapure water. The acute UC model was established by exposing the animals to 2.5% DSS in drinking water for 7 continuous days. After the successful establishment of acute UC, the DSS solution was replaced with standard tap water for another 10 days, and acute colitis healed itself.

### Preparation of TiO_2_ NPs suspension

Based on the dietary exposure data of different countries in which the daily intake of TiO_2_ in the population reached approximately 1 mg/kg/d in adults and 3 mg/kg/d in children [3], we set the exposure dosage of TiO_2_ NPs to 30, 100, 300 mg/kg/d according to uncertainty factor of 10–100 times.

TiO_2_ NPs (30, 100, 300 mg) were added to 10 ml ultrapure water to prepare TiO_2_ NP suspensions in concentrations of 3, 10, and 30 mg/ml. These three TiO_2_ NP suspensions were sonicated for 15 min before they were used every time. Considering the changes in the hydrodynamic diameters, PDI and zeta potential of TiO_2_ NPs suspension at 0–2 h post sonication (Additional file 1: Figure S1, Table S2), the gavage procedure for each group was required to be completed within 20 min after sonication. The volume of gavage in mice was calculated according to the body weight of mice, the exposure doses, and the concentration of TiO_2_ NP suspension. After intragastric administration of TiO_2_ NP suspensions, ultrapure water was administered to ensure a total volume of 0.3 ml per animal.

### Animal treatment

Healthy 8-week-old Balb/c male mice were supplied and fed under specific pathogen-free conditions by the Department of Laboratory Animal Science, Peking University Health Science Center. The mice were kept in plastic cages in a 22 ± 2 °C and 50–70% relative humidity room with a 12:12 h light–dark cycle and gave a commercial pellet diet and standard tap water *at libitum* before study. The animal experiments were carried out in accordance with the Guiding Principles in the Use of Animals in Toxicology adopted by the Society of Toxicology and received approval from the Peking University Institutional Review Board.

After five days of acclimation, 120 Balb/c mice were divided into 2 groups for 2 experiments: UC development and UC self-healing. In each part of the experiment, 60 mice were divided into 6 groups: the control (CT) group, 300 mg/kg/d TiO_2_ NPs (NPs) group, 2.5% DSS (DSS) group, 2.5% DSS + 30 mg/kg/d TiO_2_ NPs (DSS + 30 NPs) group, 2.5% DSS + 100 mg/kg/d TiO_2_ NPs (DSS + 100 NPs) group, and 2.5% DSS + 300 mg/kg/d TiO_2_ NPs (DSS + 300 NPs) group. The exposure procedure is shown briefly in Fig. 1. The UC development experiment, which lasted 7 days, was carried out during UC modeling. Mice in the CT group and NP group were normal mice, and these mice drank water freely during the 7 days. Mice in the NP group were administered 300 mg/kg/d TiO_2_ NP suspension by gavage every day. Mice in the other 4 groups were UC model mice, and these mice drank 2.5% DSS solution freely during the 7 days and were gavaged with 0, 30, 100, 300 mg/kg/d TiO_2_ NP suspension. The UC self-healing experiment, which lasted for 17 days, was carried out during the induction and self-healing period of UC. In this part of the experiment, the exposure of TiO_2_ NPs by gavage was carried out in the last 10 days, which was the stage of UC self-healing. In both experiments, mice were fasted for 1 day after the end of exposure and then harvested for further detection.

During experiments, the general conditions of the mice were observed every day, including activity, mental states, appearance and mortality. Mouse weight and water and food intake were also recorded every day. The food utilization were calculated according to the formula food utilization (%) = weight increase (g) × 100/feed consumption (g). Compared with the CT group, significantly decreased food utilization rate (Additional file 1: Figure S2) were found in the NP group on the day 15, in the DSS group on the days 9 and 15, in the DSS + 30 NPs group on the days 8, 9 and 15 days, and in the DSS + 300 NPs group on the days 9, 10 and 15, which may be connected with weight loss in the UC self-healing.

### Collection of animal samples

During mouse feeding, the feces of all mice were collected into EP tubes for fecal occult blood tests every day. On the day mice were harvested, anesthesia of mice was performed by intraperitoneal injection of 4% chloral hydrate. Blood samples were collected from the eye artery by removing the eyeball quickly. Serum and blood cells were separated by centrifuging blood samples at 4500 r/min and 4 °C for 15 min, and the supernatant was stored at − 80 °C for further detection of myeloperoxidase (MPO), T-SOD, and GSH-PX activities and cytokine expression. Then, the mice were sacrificed through cervical dislocation, and the gut was removed.

The duodenum, jejunum, ileum, and colon were carefully separated and cut in length. The intestinal content was washed out, and intestinal morphology was observed. For the colon, colonic length was detected from the cecum to the anus before cutting. After overall assessment, 1 cm pieces of each intestine were fixed in 4% formaldehyde solution for the preparation of pathological sections. The remaining intestine was stored at − 80 °C for further detection of MPO, T-SOD, and GSH-PX activities, cytokine expression and quantitative real-time polymerase chain reaction (RT-qPCR).

Other organs, including the liver, kidney, and spleen, were also separated. The caudate lobe of the liver, right kidney, and lower third of the spleen were fixed in 4% formaldehyde solution for the preparation of pathological sections. Remained organs were stored at − 80 °C.

### Histological examination

All histopathological examinations followed standard laboratory procedures as follows. Intestines, liver, kidney and spleen fixed in formaldehyde solution were embedded in paraffin and cut into 5–8 μm thick slices. The slices were stained with hematoxylin–eosin (HE). The stained slices were observed under an optical microscope and evaluated blindly by the pathologist.

The histopathological changes of the small intestine segments in the treated mice were mainly villi rupture, loss of lacteal, and inflammatory cells infiltration (Additional file 1: Figure S3). The histopathological injuries of liver, kidney, spleen was also slight, that is protein cast in renal tubules in kidney, occasionally unclear boundary between the white and red pulp in spleen, and slight inflammatory cell infiltration in the portal vein and congestion of hepatic sinus in liver (Additional file 1: Figure S4).

### Assessment of colitis activity

Colitis activity was assessed using the disease activity index (DAI) score, colonic mucosa damage index (CMDI) score, and histological changes in the colon following the methods described in our published work [53].

The DAI score was calculated according to the weight loss rate, fecal characteristics and fecal occult blood (Additional file 1: Table S3). The CMDI score was evaluated based on colonic adhesion and ulcer formation and inflammation (Additional file 1: Table S4). Histological changes in the colon were evaluated by colonic length, colonic mucosal thickness, and colonic histopathological score. The colon was straightened slightly after the colon was removed from the mice, and colonic length was detected from the cecum to the anus using a ruler. Three mice were chosen from each group to prepare pathological sections. Three visual fields under a low-power lens were randomly selected from colon sections of each mouse. Then, 3 points in each visual field were randomly selected to measure the vertical distance from the tip of the mucosa to the muscularis mucosa. The mean value of 3 points in each field was used as the mucosal thickness of the field, and the mean value of 3 fields was used as the mucosal thickness of the mice. Finally, the mean value of the mucosal thickness of each group was used as the final result. The colonic histopathological score was the sum of the scores of epithelial change and inflammatory cell infiltration, as listed in Additional file 1: Table S5. The histopathological changes of the colon segments of mice were present in Fig. 3, including the crypt and goblet cell loss and inflammatory infiltration.

### Evaluation of inflammatory response level

Following the published study [54], MPO activity and cytokine expression were detected in this section. MPO activity reflects the degree of infiltration of neutrophils in colon tissues. According to the manufacturer’s instructions of Nanjing Jiancheng Bioengineering Institute, China, colonic homogenate was prepared, and MPO activity in colon tissues was detected by the o-anise staining method. IL-1β and IL-18 expression in the colon and serum was detected using enzyme-linked immunosorbent assay (ELISA) according to the manufacturer’s instructions.

### Oxidative stress tests

To detect ROS production and enteritis in UC mice induced by DSS, in vivo imaging was performed using chemiluminescence based on luminol reaction to observe the production of ROS in the abdomen of mice [55]. During the UC development experiment, 3 mice from each group were randomly selected in the CT, DSS, and DSS + 300 NP groups and anesthetized using 1.5%-2.5% isoflurane gas. A 20 mg/kg 8-amino-5-chloro-7-phenylpyrido(3,4-d) pyridazine-1,4(2H,3H) dione (L-012) chemiluminescent probe was injected intraperitoneally, and mice were imaged using the IVIS Spectrum live animal imaging system after 5 min. The L-012 chemiluminescent probe for injection was dissolved in PBS before use and stored at 4 °C away from light. The region of interest (ROI) was used to quantify the luminous intensity of the mouse abdomen.

The stored colon was thawed, and 0.15–0.2 g was taken and used to prepare a 10% colonic homogenate using 0.86% (mass fraction) NaCl solution. The colonic homogenate was centrifuged at 4000 r/min and 4 °C for 15 min, and the supernatant was retained. The protein content of colonic homogenate was detected by the Bradford method. To determine the status of oxidative stress in mice, T-SOD, GSH-PX, and catalase (CAT) activities and malonaldehyde (MDA) content in colonic homogenate and T-SOD and GSH-PX activities and MDA content in serum were detected according to the manufacturer’s instructions**.**

#### RT–qPCR analysis

TXNIP, cysteinyl aspartate specific proteinase 1 (caspase-1), and IL-1β mRNA expression in the colon was detected using RT–qPCR. Total RNA of colonic tissues was isolated using TRIzol reagent (Sangon Biotech, Shanghai, China), and cDNA was synthesized using HiScript II Q RT SuperMix for qPCR (Vazyme Biotech Co., Ltd., Nanjing, China) according to the instructions. The internal control was glyceraldehyde-3-phosphate dehydrogenase (GAPDH). RT–PCR was performed using AceQ qPCR SYBR Green Master Mix. A mixed PCR system was prepared containing 10 μl qPCR SYBR Green Master mix (Vazyme Biotech Co., Ltd, Nanjing, China), 0.4 μl PCR forward primer, 0.4 μl PCR reverse primer (Invitrogen, USA), 3 μl ddH_2_O, moderate cDNA that made concentrate of final reaction system 0.2 μmol/L, and moderate cDNA that made volume of final reaction system 20 μl. All reactions were performed in triplicate. Then, the reactions were performed in a real-time fluorescence quantitative PCR instrument. Relative changes in gene expression were determined using the 2^–ΔΔCt^ method (ΔΔCt = (Ct^A2^ − Ct^B2^) − (Ct^A1^ − Ct^B1^). Ct^A1^ and Ct^A2^ were the Ct values of the target genes of sample 1 and sample 2, respectively, and Ct^B1^ and Ct^B2^ were the Ct values of the internal reference genes of sample 1 and sample 2, respectively. The primer sequences were as follows:

- caspase-1 forward: ACAAGGCACGGGACCTATG,
  - reverse: TCCCAGTCAGTCCTGGAAATG
- IL-1β forward: TTCAGGCAGGCAGTATCACTC,
  - reverse: GAAGGTCCACGGGAAAGACAC
- TXNIP forward: GGCCGGACGGGTAATAGTG,
  - reverse: AGCGCAAGTAGTCCAAAGTCT
- GAPDH forward: AGGTCGGTGTGAACGGATTTG,
  - reverse: GGGGTCGTTGATGGCAACA

#### Statistical analysis

SPSS 20.0 was used to carry out the statistical analysis. Data that exhibited a normal distribution according to the K-S test are expressed as the mean value ± standard deviation ($\overline{x }$ ± SD). The variance homogeneity test and one-way ANOVA were used to compare variance among groups. Differences between two groups were analyzed by LSD-t test if the variance was homogeneous and analyzed by Games-Howell test if the variance was not homogeneous. P < 0.05 was considered statistically significant.

## Supplementary Information

**Additional file 1.** Oral intake of titanium dioxide nanoparticles affect the course and prognosis of ulcerative colitis in mice: Involvement of the ROS-TXNIP-NLRP3 inflammasome pathway.

## Data Availability

The datasets used and/or analyzed during the current study are available from the corresponding author on reasonable request.

## Abbreviations

TiO_2_: Titanium dioxide
UC: Ulcerative colitis
DSS: Dextran sodium sulfate
DAI: Disease activity index
CMDI: Colonic mucosa damage index
ROS: Reactive oxygen species
T-SOD: Total superoxide dismutase
GSH-PX: Glutathione peroxidase
CAT: Catalase
TXNIP: Thioredoxin interacting protein
NLRP3: NLR family pyrin domain containing 3
FDA: Food and drug administration
NPs: Nanoparticles
GIT: Gastrointestinal tract
M cells: Microfold cells
IBD: Inflammatory bowel disease
CD: Crohn’s disease
GSH-PX: Glutathione peroxidase
LP: Lamina propria
CT: Control
MPO: Myeloperoxidase
TNF-γ: Tumor necrosis factor γ
GSH: Glutathione
GSSG: Glutathione
BET: Brunauer–Emmett–Teller
AGJ: Artificial gastric juice
AIJ: Artificial intestinal juice
HE: Hematoxylin–eosin
DAI: Disease activity index
CMDI: Colonic mucosa damage index
MDA: Malonaldehyde
GAPDH: Glyceraldehyde-3-phosphate dehydrogenase
