# Supplementary material for: Oral intake of titanium dioxide nanoparticles affect the course and prognosis of ulcerative colitis in mice: involvement of the ROS-TXNIP-NLRP3 inflammasome pathway
Source: Part Fibre Toxicol. 2023 Jun 22;20:24. doi: 10.1186/s12989-023-00535-9 (PMC10288682; doi:10.1186/s12989-023-00535-9)
Supplement: Supplementary file 1 — Additional file 1. Oral intake of titanium dioxide nanoparticles affect the course and prognosis of ulcerative colitis in mice: Involvement of the ROS-TXNIP-NLRP3 inflammasome pathway. [file 12989_2023_535_MOESM1_ESM.docx]

Supporting Information

**Oral Intake of Titanium Dioxide Nanoparticles Affect the Course and Prognosis of Ulcerative Colitis in Mice: Involvement of the ROS-TXNIP-NLRP3 Inflammasome Pathway**

*Shumin Duan^1^, Hongbo Wang^1^, Yanjun Gao^1^, Xiang Wang^2^, Lizhi Lyu^1^, Yun Wang^1^**

^1^ Department of Occupational and Environmental Health Sciences, School of Public Health, Peking University, Beijing 100191, P.R. China; Beijing Key Laboratory of Toxicological Research and Risk Assessment for Food Safety, Peking University, Beijing 100191, P.R. China

^2^ California NanoSystems Institute, University of California, Los Angeles, California 90095, United States

* Corresponding Author’s Email: wangyun@bjmu.edu.cn

| **Table S1 Number of mouse deaths during the experiment (*n*=10)** | | |
| --- | --- | --- |
| Group | Number of mice deaths in UC developing | Number of mice deaths in UC self-healing |
| CT | 0 | 0 |
| NPs | 0 | 0 |
| DSS | 1 | 0 |
| DSS+30 NPs | 0 | 2 |
| DSS+100 NPs | 2 | 0 |
| DSS+300 NPs | 1 | 1 |

Table S2 The zeta potential, hydrodynamic diameters and polydispersity index (PDI) of TiO_2_ in ultrapure water, artificial gastric juice (AGJ) and artificial intestinal juice (AIJ). ($\bar{x}$±*s, n=3*)

| Testing time | Concentration of TiO_2_ | Solvent | Zeta potential (mV) | Hydrodynamic diameters (d.nm) | PDI |
| --- | --- | --- | --- | --- | --- |
| 0 h post sonication | 3 mg/ml | Ultrapure water | 12.3±0.69 | 1079±23 | 0.25±0.19 |
|  |  | AGJ | 2.77±1.11 | 2298±24 | 0.44±0.03 |
|  |  | AIJ | -11.4±1.1 | 3207±103 | 0.30±0.03 |
|  | 10 mg/ml | Ultrapure water | 12.3±0.26 | 891±22 | 0.41±0.02 |
|  |  | AGJ | -0.01±6.20 | 1227±34 | 0.50±0.08 |
|  |  | AIJ | 4.42±6.29 | 8080±2611 | 0.92±0.15 |
|  | 30 mg/ml | Ultrapure water | -0.79±0.50 | 1760±472 | 0.98±0.38 |
|  |  | AGJ | 4.04±1.21 | 1355±11 | 0.51±0.20 |
|  |  | AIJ | 1.16±1.75 | 6446±1343 | 0.96±0.06 |
| 1 h post sonication | 3 mg/ml | Ultrapure water | -0.12±0.23 | 926±25 | 0.46±0.33 |
|  |  | AGJ | 17.6±0.23 | 932±44 | 0.26±0.02 |
|  |  | AIJ | 20.0±0.25 | 1322±58 | 0.12±0.09 |
|  | 10 mg/ml | Ultrapure water | -5.2±0.37 | 1116±101 | 0.16±0.03 |
|  |  | AGJ | 13.0±0.36 | 1437±40 | 0.25±0.07 |
|  |  | AIJ | 18.0±0.85 | 1538±62 | 0.30±0.07 |
|  | 30 mg/ml | Ultrapure water | 10.5±0.35 | 918±109 | 0.27±0.01 |
|  |  | AGJ | 5.76±0.49 | 865.7±18 | 0.26±0.01 |
|  |  | AIJ | 11.2±1.5 | 1389±51 | 0.37±0.01 |
| 2 h post sonication | 3 mg/ml | Ultrapure water | 11.7±0.15 | 883±3 | 0.23±0.01 |
|  |  | AGJ | 19.5±0.6 | 792±2 | 0.14±0.03 |
|  |  | AIJ | 19.0±2.0 | 1092±17 | 0.22±0.08 |
|  | 10 mg/ml | Ultrapure water | 12.3±0.26 | 996±59 | 0.25±0.06 |
|  |  | AGJ | 12.9±0.15 | 1194±31 | 0.22±0.04 |
|  |  | AIJ | 19.6±1.00 | 1424±149 | 0.31±0.02 |
|  | 30 mg/ml | Ultrapure water | 10.2±0.36 | 673±30 | 0.35±0.06 |
|  |  | AGJ | 11.6±0.95 | 743±11 | 0.27±0.06 |
|  |  | AIJ | 14.1±0.75 | 1500±225 | 0.39±0.15 |

| **Table S3 DAI score****（*n*=120）** | | | |
| --- | --- | --- | --- |
| score | weight loss rate (%) | fecal character | fecal occult blood |
| 0 | 0 | normal | negative |
| 1 | 1-5 | soft stool | + |
| 2 | 6-10 | loose stool | ++ |
| 3 | 11-15 |  | +++ |
| 4 | >15 | watery stool | ++++ |

| **Table S4 CMDI score（*n*=120）** | |
| --- | --- |
| score | gross morphology |
| colonic adhesion | |
| 0 | no |
| 1 | mild or the colon is easy to be separated from other tissues |
| 2 | severe |
| ulcer formation and inflammation | |
| 0 | normal |
| 1 | local hyperemia, no ulcer |
| 2 | the ulcer is not accompanied by hyperemia, or intestinal wall thickening |
| 3 | 1 ulcer with inflammation |
| 4 | >=2 ulcers with inflammation |
| 5 | ulcers and/or inflammation >1cm |
| >6 | ulcers and/or inflammation >2cm，the score was increased by 1 for each 1cm increase in lesion range |

| **Table S5 Colonic histopathological score（*n*=36）** | |
| --- | --- |
| score | assessment |
| Epithelial change | |
| 0 | normal |
| 1 | goblet cell loss |
| 2 | large areas of goblet cell loss |
| 3 | crypt loss |
| 4 | Large areas of crypt loss |
| Inflammatory cell infiltration | |
| 0 | no infiltration |
| 1 | infiltrate into the basal layer of crypt |
| 2 | infiltrate into the muscularis mucosa |
| 3 | infiltrate into the muscularis mucosa, accompanied by thickening of mucosa and marked edema |
| 4 | infiltration reaches the submucosa |


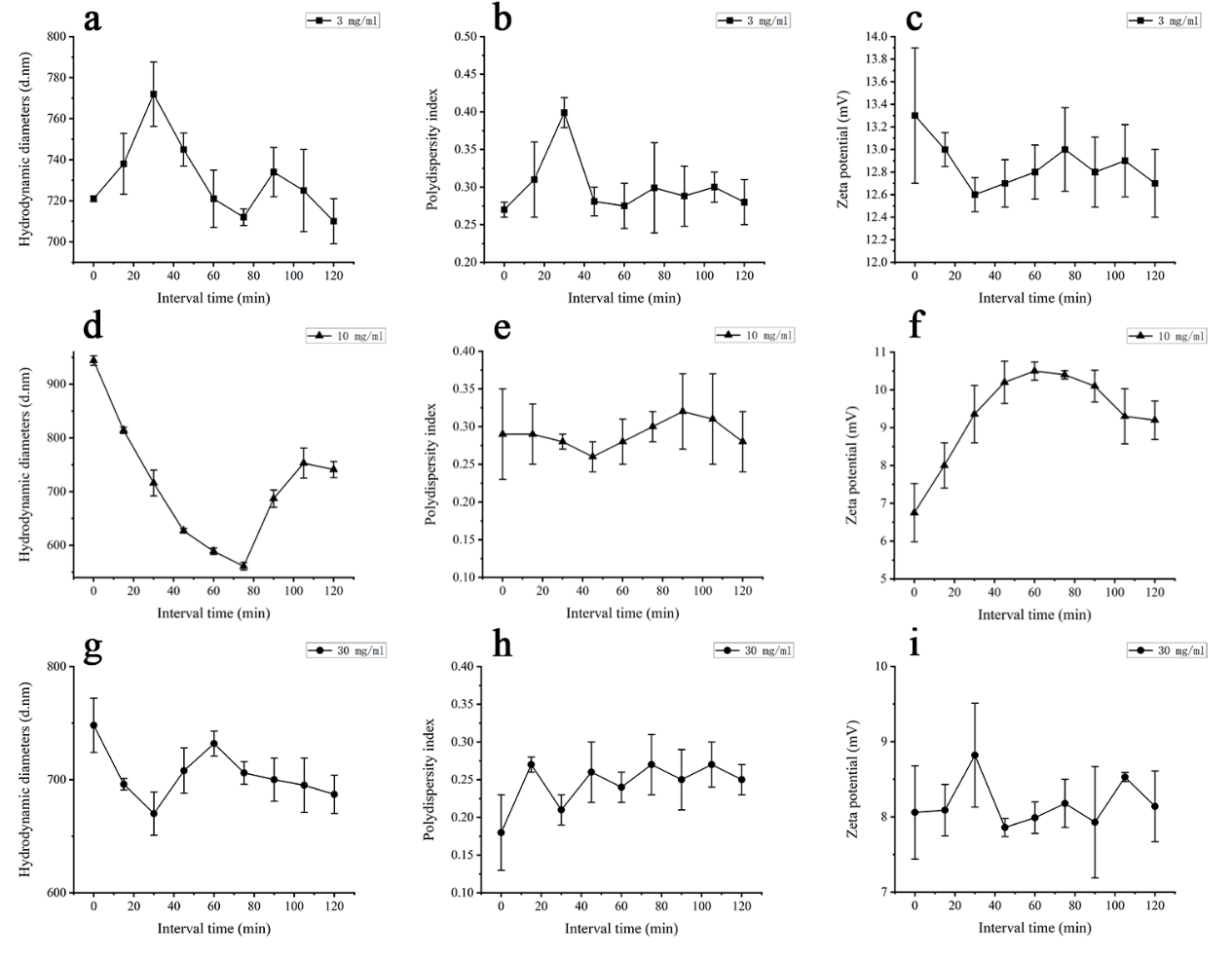


**Figure S1** The variation of hydrodynamic diameters, polydispersity index (PDI) and zeta potential of 3 mg/ml (a, b and c), 10 mg/ml (d, e and f) and 30 mg/ml (g, h and i) TiO_2_ in ultrapure water with the interval time between tests after ultrasonic treatment.


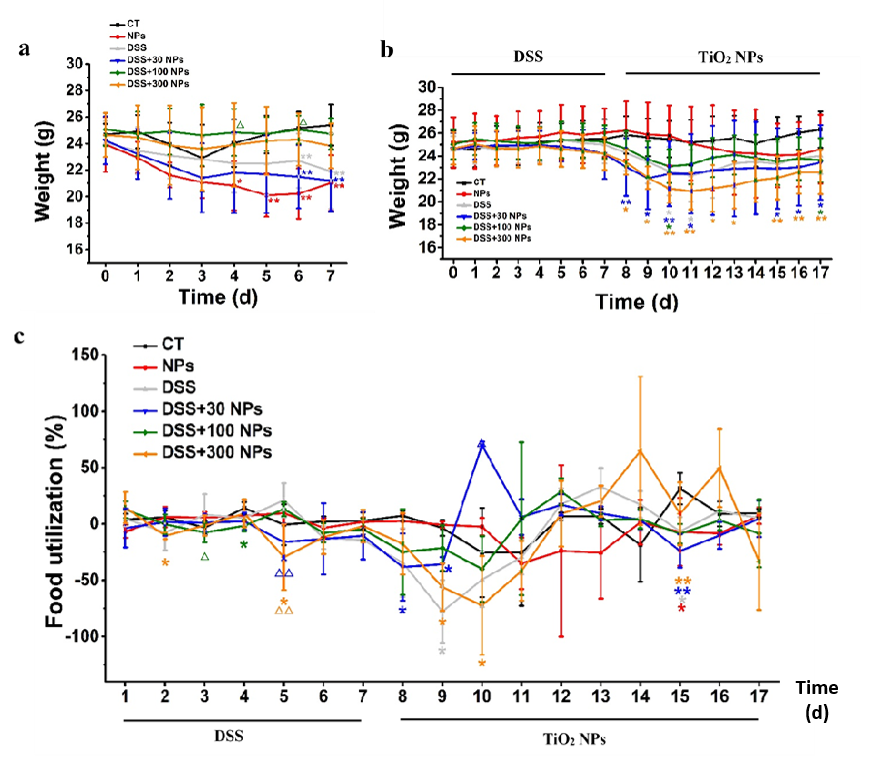


**Figure S2** Change of food utilization in the experiment of UC self-healing (*n* =10，$\bar{x}$±*s*). * p<0.05, ** p<0.01, compared with CT group; △ P<0.05, △△ P<0.01, compared with DSS group.


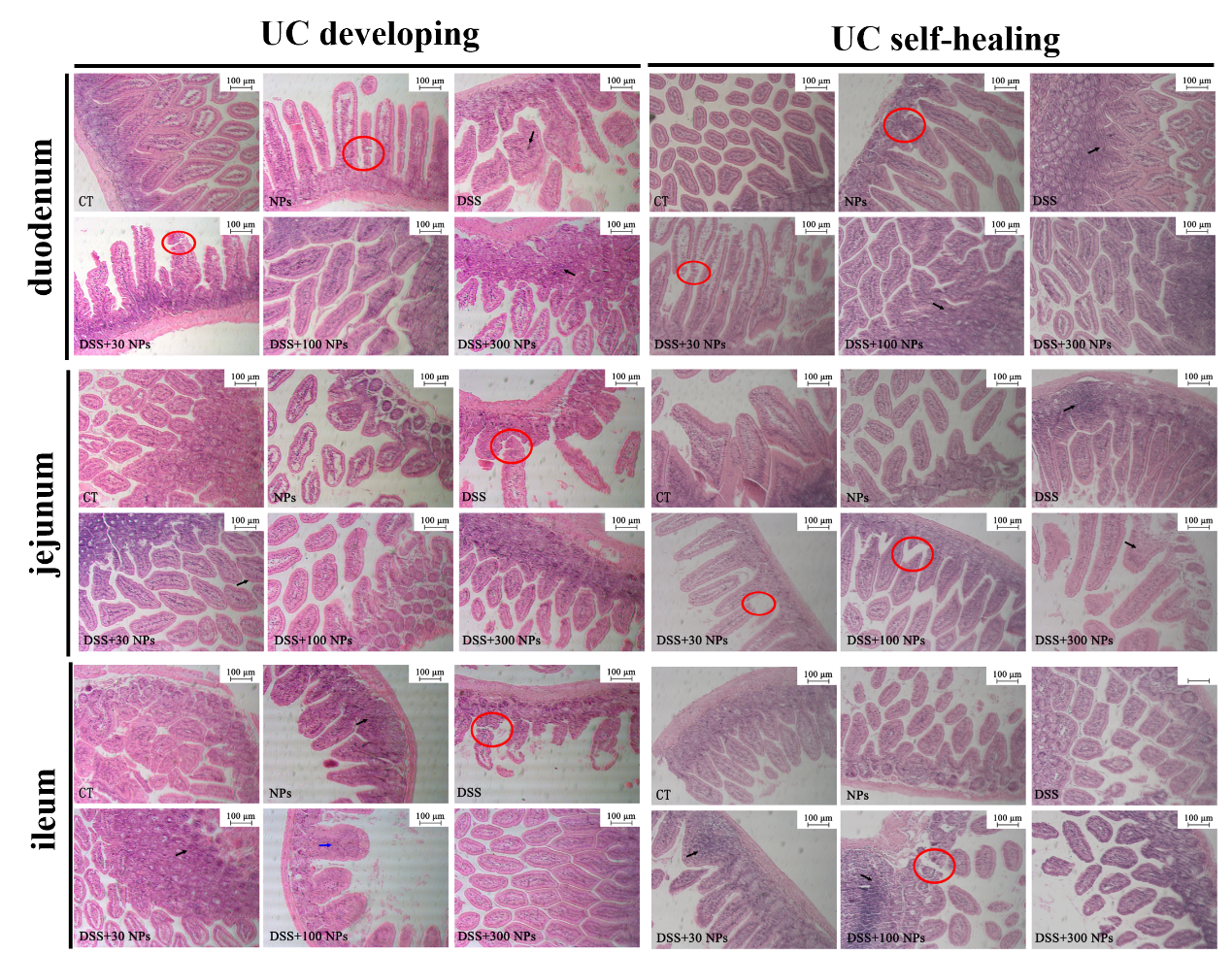


**Figure S3** Histopathological changes in the small intestine of mice in the experiment of UC developing and UC self-healing. Red circle means rupture of villi, black arrow means inflammatory cells infiltration, and blue arrow means loss of lacteal in villi.


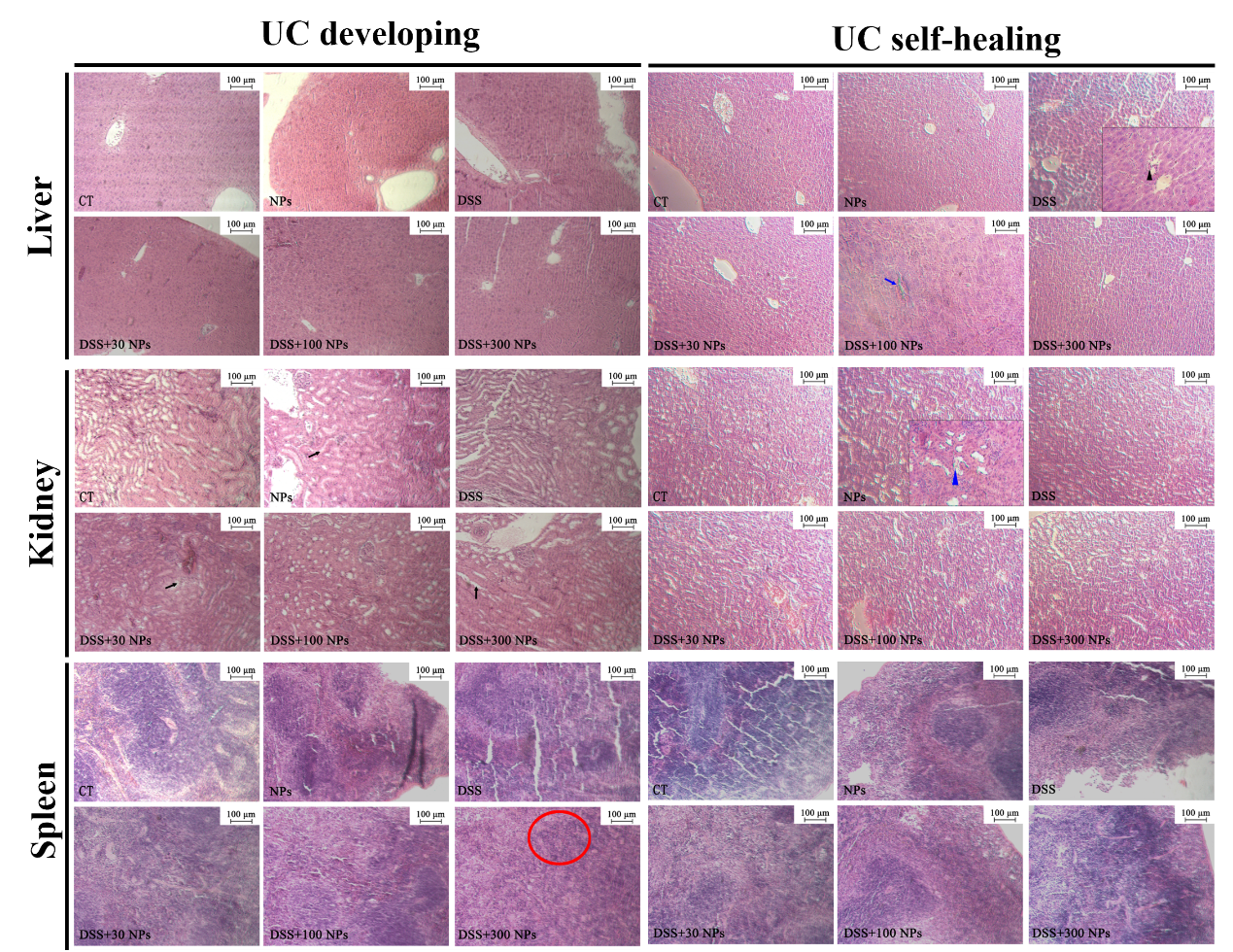


**Figure S4** Histopathological changes in liver, kidney, spleen of mice in the experiment of UC developing and UC self-healing. Black triangle means inflammatory cell infiltration, blue triangle means renal tubules shedding, black arrow means protein cast, blue arrow means hepatic sinus congestion, and red circle means that the boundary of the white and red medulla is unclear.
